# Hello Darkness, My Old Friend: Moderating a Random Intercept Cross-lagged Panel Model of Loneliness and Symptoms of Anxiety and Depression

**DOI:** 10.1007/s10802-022-00995-1

**Published:** 2022-11-23

**Authors:** Sara Madeleine Kristensen, Helga Bjørnøy Urke, Torill Bogsnes Larsen, Anne Grete Danielsen

**Affiliations:** 1 Department of Health Promotion and Development, University of Bergen, Bergen, 5009 Norway; 2 Department of Education, University of Bergen, Bergen, 5020 Norway

**Keywords:** Symptoms of anxiety and depression, Loneliness, Random intercept cross-lagged panel model, Gender, Social self-efficacy

## Abstract

The present study investigated the trait- and state-like associations between loneliness and symptoms of anxiety and depression during three years in middle to late adolescence. The moderating effect of gender and social self-efficacy was examined on the hypothesised model. The sample consisted of 1508 Norwegian upper secondary school students (61% female; mean age at T1 = 16.33; 52.9% high socioeconomic position; 70.6% Norwegian-born). We found 1) strong and positive trait- and state-like associations between loneliness and symptoms of anxiety and depression, 2) that anxiety and depressive symptoms consistently predicted later loneliness but not the other way around, 3) that gender moderated parts of the state-like associations between loneliness and symptoms of anxiety and depression, and 4) that social self-efficacy had no moderating effect on the longitudinal relationship between loneliness and anxiety and depressive symptoms. The present study might inform future research, theory development, and intervention strategies in middle to late adolescent samples.

**Supplementary Information:**

The online version contains supplementary material available at 10.1007/s10802-022-00995-1.

## Introduction

Loneliness (i.e., a perception of being socially isolated: Cacioppo & Hawkley, 2009; Hawkley & Cacioppo, 2010) is a relatively common and temporary experience throughout our lives, which tends to be prevalent in adolescence (Goossens, 2018; Laursen & Hartl, 2013; Qualter et al., 2015). The presence of loneliness during the adolescent period is mainly due to the social reorientation process that adolescents undergo, which is characterised by several social challenges (Goossens, 2018). Overcoming these challenges and connecting with peers become increasingly salient throughout adolescence. For instance, a lack of intimate friendships is more associated with depression in adolescence than in children (Buhrmester, 1990) – it seems that friendship quality becomes more important than quantity during the adolescent years (Qualter et al., 2015). Because symptoms of anxiety and depression also increase considerably during this period (Hankin et al., 1998; Zahn–Waxler et al., 2000), mid-late adolescence could represent a crucial developmental life stage to investigate how perceived social isolation and symptoms of anxiety and depression are associated within individuals (Goossens, 2006; Hankin et al., 1998).

The assumed reciprocal relationship between loneliness and symptoms of anxiety and depression in adolescence has gained increased focus recently (e.g., Danneel et al., 2019; Lasgaard et al., 2011; Vanhalst et al., 2012). Danneel et al. (2019) found that loneliness and social anxiety symptoms were reciprocal in nature, while depressive symptoms predicted later loneliness and not the other way around. Similarly, Lasgaard et al. (2011) established that depressive symptoms were associated with subsequent loneliness but not vice versa. Vanhalst et al. (2012) showed that loneliness and depressive symptoms were reciprocally related across five years in adolescence. Although this research has increased our understanding of the prospective associations between loneliness, anxiety, and depression, there is a noteworthy shortcoming in the mentioned studies. Namely, they have not disaggregated within-person effects from between-person effects, making the temporal associations somewhat inaccurate (Hamaker et al., 2015) and the theoretical impacts questionable (Curran & Bauer, 2011).

When separating within-person effects from between-person effects, people’s personal norm of a factor (i.e., their trait-like level across the study’s duration) is separated from the deviations individuals experience at each measurement occasion (i.e., their state-like level at each time point that diverges from their personal norm) (Hamaker et al., 2015). By using the deviating state-like factors to investigate the association between loneliness and symptoms of anxiety and depression over time, we can increase our understanding of the true nature of the temporal relations; it can allow us to evaluate central theoretical assumptions and thereby be relevant for future intervention strategies on to improve mental health and decrease perceived social isolation.

We use empirical findings from adolescent samples and combine several theoretical assumptions to investigate the within-person association between loneliness and symptoms of anxiety and depression in mid-late adolescence. Specifically, we 1) incorporate both anxiety and depressive symptoms in one construct based on the rationales of the cumulative interpersonal risk model by Epkins and Heckler (2011), 2) use the evolutionary theory of loneliness (Cacioppo & Cacioppo, 2018) to explain how loneliness may function as an antecedent of symptoms of anxiety and depression, 3) use the interpersonal theory of depression (Coyne, 1976) to illustrate how symptoms of anxiety and depression might lead to loneliness, and 4) use the developmentally based interpersonal model of depression by Rudolph et al. (2008) to hypothesise moderating effects on the longitudinal association between loneliness and symptoms of anxiety and depression.

### Developmental Processes of Loneliness and Symptoms of Anxiety and Depression

#### The Evolutionary Theory of Loneliness

According to the evolutionary theory of loneliness by Cacioppo and Cacioppo (2018), perceived social isolation (i.e., loneliness) functions as a warning signal that brings attention to the possible deterioration of the body. Humans are regarded as inherently social beings (Cacioppo & Patrick, 2008), and when the innate, strong desire to connect with others is thwarted, people become motivated to fix the perceived deficiencies in their social relationships and thus avoid the negative emotions (Cacioppo & Hawkley, 2009). Paradoxically, people can also be motivated to be alert and avoid possible social dangers to ensure their self-preservation (Cacioppo & Cacioppo, 2018), or in other words, socially withdraw. The evolutionary theory proposes that the signal to self-preserve (i.e., feeling lonely) triggers several behavioural and physical adjustments to manage and deal with faulty social relations, thus avoiding premature mortality. One adjustment is increased depressive symptomatology (Cacioppo & Cacioppo, 2018). Several studies have found support for this theorised effect (for an overview, see e.g., Loades et al., 2020).

#### The Interpersonal Theory of Depression

The interpersonal theory of depression (Coyne, 1976) argues that depressed and depressed-prone individuals have certain characteristics and behave in manners that impede social relationships by eliciting negative reactions from others (Giesler et al., 1996; Gotlib & Hammen, 1992; Joiner et al., 1992; Swann et al., 1992) and produce interpersonal stress and conflict (Hammen, 2020; Rudolph et al., 2000). For example, depressed people may attempt to decrease feelings of personal guilt and low self-worth through excessive reassurance from people they are close to (Coyne, 1976). Initially, people might offer support. However, because the depressed person is uncertain about the genuineness, they continue to demand reassurances which causes others to become agitated and reject them (Starr & Davila, 2008). Indeed, studies indicate that, compared to non-depressed people, depressed individuals are increasingly likely to experience social dysfunctions (Gotlib & Lee, 1989), less enjoyment and intimacy from social interactions (Nezlek et al., 2000), and withdraw from social interactions (Schaefer et al., 2011).

#### Two Models of Relevance

The cumulative interpersonal risk model (Epkins & Heckler, 2011) argues that although symptoms of anxiety (particularly social anxiety) and depression have similar etiological influences, most researchers overlook the considerable overlap in or comorbidity of anxiety and depressive symptoms. For instance, recent findings imply that certain genomes are related to the co-occurrence of anxiety and depressive symptoms (Jami et al., 2022). Thus, in this study, we combine symptoms of anxiety and depression in one construct, examining the shared and overlapping risk factors and/or consequences that symptoms of anxiety and depression pose in relation to loneliness during mid-late adolescence. This might advance research that is theoretically and empirically relevant to prevention and treatment (Epkins & Heckler, 2011).

The developmentally based interpersonal model of depression (Rudolph et al., 2008, p. 80; see Appendix A for details) suggests that gender and social cognitive factors (e.g., social self-efficacy) might moderate the extent to which relationship disturbances heighten the risk for depression (Hankin & Abela, 2005), as well as the extent to which depression influences interpersonal functioning (Coyne et al., 1998). Empirical findings indicate that the association between loneliness and symptoms of anxiety and depression could be stronger for girls than for boys (Chang, 2018; Rudolph et al., 2008). Girls, compared to boys, are increasingly likely to define themselves based on their interrelationships, be more dependent on social connections, prioritise goals in line with their relationships, and worry about others’ evaluations of them (Rose & Rudolph, 2006). Furthermore, girls tend to increase in depressive symptoms due to their reactions to interpersonal stress, such as rumination (Rose & Rudolph, 2006).

People with a cognitive vulnerability are increasingly likely to experience a pattern of information processing that is inherently negatively biased, facilitating a descent into depression (Abela & Hankin, 2008). Social self-efficacy (i.e., social capability beliefs; Bandura, 1977, 1997) may function as a vulnerability/protective factor in the association between loneliness and symptoms of anxiety and depression. It is assumed that socially efficacious people pursue and maintain social connections that help the individual be in control of difficult situations and dampen the effect of negative life events. In contrast, people with low social self-efficacy have increased vulnerability to depression through social isolation (Bandura, 1994). Indeed, social self-efficacy is negatively associated with loneliness (Hermann & Betz, 2006; Tsai et al., 2017; Wei et al., 2005) and symptoms of anxiety and depression (Hermann & Betz, 2004; Kristensen et al., 2021; McFarlane et al., 1995) in adolescence and young adulthood.

### Study Aims

Although the relationship between loneliness and symptoms of anxiety and depression in adolescence is gaining increased focus (Goossens, 2018), there is a gap in the research literature. The association between loneliness and anxiety and depressive symptoms has largely, perhaps only, been investigated on a between-person level, despite the fact that the relationship between the two should arguably be examined on a within-person level. That is, the temporal association between loneliness and symptoms of anxiety and depression might only make sense if the experienced levels in both constructs are relative to individuals’ own normative level (i.e., how deviations in one construct are related to deviations in the other construct within individuals over time).

**Fig. 1:**
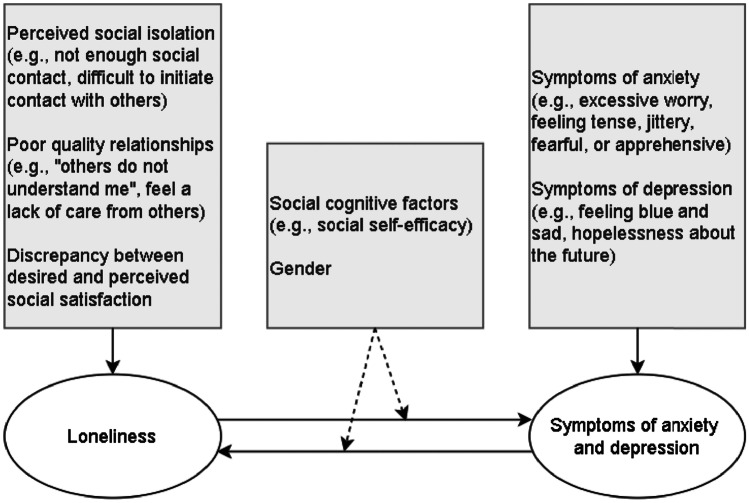
Hypothesised Relationship Between Loneliness and Symptoms of Anxiety and Depression. *Note*. The model is based on theoretical assumptions in ETL and rationales from the interpersonal model of youth depression (Rudolph et al., 2008, p. 80) and the cumulative interpersonal risk model (Epkins & Heckler, 2011). Dashed lines represent moderating effects

Our study aims to fill the abovementioned research gap. We use the evolutionary theory of loneliness (Cacioppo & Cacioppo, 2018), the interpersonal theory of depression (Coyne, 1976), the cumulative interpersonal risk model (Epkins & Heckler, 2011), and the developmentally based interpersonal model of depression (Rudolph et al., 2008) to create a hypothetical model of loneliness and symptoms of anxiety and depression, possibly moderated by gender or social self-efficacy. The model is presented in Fig. 1. Although little or no research has been done on the within-person associations of the study’s variables, we use the abovementioned theoretical frameworks and models to inform the following hypotheses:

1. Loneliness and symptoms of anxiety and depression are positively related on a trait- and state level.
2. Loneliness is positively associated with subsequent symptoms of anxiety and depression at the within-person level.
3. Symptoms of anxiety and depression are positively associated with subsequent loneliness at the within-person level.
4. Gender moderates the association between loneliness and symptoms of anxiety and depression (i.e., the association is stronger for girls compared to boys).
5. Social self-efficacy moderates the association between loneliness and symptoms of anxiety and depression (i.e., the association is stronger for people with low social self-efficacy compared to people with high social self-efficacy).

## Methods

### Procedure

The COMPLETE project (Larsen et al., 2018) followed a sample of Norwegian adolescents from approximate age 15 to 19. The project is a randomised controlled trial with two intervention groups and one control group. There were six schools each in the intervention group and four schools in the control group. All general education programme students who enrolled in the first year of upper secondary school (a three-year education) in August 2016 in the selected schools were asked to participate in the study. The students who agreed answered a questionnaire shortly after and were invited to respond to the same questionnaire again in March, nearing the end of the school year, in 2017, 2018, and 2019. Students under the age of 16 at baseline needed parental/guardian consent to participate in the study. Individuals who did not provide this consent were invited to participate again at the following measurement occasion – all students were above the age of 16 at this time point. The study was approved by the Norwegian Centre for Research Data (NSD), and the participants received written and oral information about the study ahead of participation. Data was gathered on school grounds physically by research members in the project.

### Participants

The sample consisted of 1508 adolescents (see Missing Data section for more information on response rates), wherein 60.7% (N = 916) were girls, and 39.3% (N = 592) were boys. The mean age at the first measurement occasion was 16.33 (*SD* = 0.62). Concerning the ethnicity of the participants, 70.6% (N = 1065) were Norwegian-born, 5.5% (N = 83) were non-ethnic Norwegian, and 23.9% (N = 360) did not answer the question. A median split of perceived family wealth showed that 52.9% (N = 797) perceived their family as being in a high socioeconomic position, while 22.5% (N = 340) perceived their family as being in a low socioeconomic position and 24.6% (N = 371) did not answer the question.

### Measurements

#### Loneliness

Loneliness was measured using a slightly modified six-item short form of the UCLA loneliness scale, developed for population-based studies in Western Norway (Kraft & Loeb, 1997; Mittelmark et al., 2004). An example indicator is ‘I feel lonely even when I am around other people’. The participants rated their answers on a scale ranging from 1 ‘Not at all true’ to 5 ‘Very true’. The adapted short form has shown adequate reliability, with Cronbach’s alpha values above 0.77 (Mittelmark et al., 2004).

#### Symptoms of Anxiety and Depression

To measure anxiety and depressive symptoms, a short Norwegian version of the Symptom Check List-90-R (SCL-5; Tambs & Moum, 1993) was used, with items from the anxiety and depression subscales. The participants were asked to rate how bothered or distressed they had felt during the last 14 days on a scale ranging from 1 ‘not at all’ to 4 ‘very much’. Indicator examples of anxiety and depression are ‘nervousness or shakiness inside’ and ‘feeling hopeless about the future’, respectively. Studies have found the Cronbach’s reliability of the SCL-5 to be higher than 0.83 (Gjerde et al., 2011; Skrove et al., 2013; Strand et al., 2003; Tambs & Moum, 1993). Of note, this scale is not a clinical assessment of or diagnostic tool for anxiety and depression but instead a measure of *symptoms* of mixed anxiety and depression (Siqveland et al., 2016). The cut-off value to best predict the presence of mental disorders and/or belonging to a high-risk group is 2.0 for the SCL-5 (Strand et al., 2003).

#### Social Self-efficacy

We used the social subscale from the Self-efficacy Questionnaire for Children (SEQ-C: Muris, 2001) to measure social self-efficacy. The scale was adapted to fit the adolescent age group, that is, wordings like ‘children’ were replaced with ‘peers’ and so on. The social SEC-Q consists of seven indicators that participants rated on a scale ranging from 1 ‘not at all’ to 5 ‘very well’. A sample item is ‘How well can you become friends with peers?’. Previous research has found Cronbach’s alpha values above 0.81 in adolescent samples (Minter & Pritzker, 2015; Muris, 2001, 2002). Because social self-efficacy was tested as a moderator, we created a dummy variable by doing a median split on the personal mean level of social self-efficacy across all measurement waves. Thus, adolescents were, on average, either in the low (coded as 0) or high (coded as 1) social self-efficacy group.

#### Gender

Gender was coded as 0 (boys) and 1 (girls).

#### Control Variables

We included several time-invariant covariates in the model. The participant’s socioeconomic position was assessed by a question on how well off economically they perceived their family to be (Iversen & Holsen, 2008). Individuals rated their perceived family wealth on a scale ranging from 1 (not at all well off) to 5 (very well off). Baseline socioeconomic position was dummy coded as 0 (low) and 1 (high) by a median split. Ethnicity was coded as 0 (Norwegian-born) and 1 (non-ethnic Norwegian). The two dummy variables for intervention conditions were coded as 0 (control group) and 1 (intervention condition).

### Data Analyses

#### Preliminary Analyses

Initially, we investigated the omega reliability and longitudinal measurement invariance of the study’s constructs. Next, we followed the procedure by Snedecor and Cochran (1980) to investigate the difference in correlation coefficients between subgroups across time. We used SPSS version 25 for data cleaning and correlation analysis and M*plus* version 8 (Muthén & Muthén, 1998–2017) for structural equation modelling. We used the following criteria to evaluate if the structural equation models achieved acceptable fit: CFI (> 0.90), RMSEA (< 0.08), and SRMR (< 0.08) (Hu & Bentler, 1999). When performing longitudinal measurement invariance testing, we used the effects-coding approach by (Little et al., 2006). Thus, the latent factors’ means and variances were constrained to 0.0 and 1.0, respectively. We specified configural models for the loneliness and symptoms of anxiety and depression constructs and applied factor loading constraints to establish weak (metric) measurement invariance across time and the moderation subgroups. Acceptable differences between the nested models and the comparison models were evaluated using the following criteria: ΔCFI < 0.010, ΔRMSEA < 0.015, and ΔSRMR < 0.030 (Chen, 2007). The invariance constraints were retained for further modelling. For space considerations, measurement invariance results are presented in the appendices (see Appendix B for details).

#### Primary Analyses

For the main analysis, we specified a random intercept cross-lagged panel model (RI-CLPM) between loneliness and symptoms of anxiety and depression using maximum likelihood estimation. This was achieved by creating corresponding latent factors for each first-order latent factor at all time points (four variables in each construct), with factor loading constrained to 1 and a random intercept for each construct, regressed by all first-order latent factors, with factor loadings constrained to 1 (Hamaker, 2018; Hamaker et al., 2015; Mulder & Hamaker, 2021). The variance of the first-order latent variables was constrained to 0.0 to ensure all variance is captured by the between-person variables (intercepts) and within-person variables. Next, we included gender, social self-efficacy, socioeconomic position, ethnicity, and intervention conditions as time-invariant covariates regressed by the random intercepts. Lastly, we investigated whether the correlation and regression coefficients in the RI-CLPM were time-invariant by constraining the within-person correlation coefficients and autoregressive- and cross-lagged regression coefficients to be equal over time and comparing the model fit of the nested model to the comparison model. A significant deterioration of model fit would indicate that the effects were not invariant over time and the constraints would be removed for further modelling.

Next, we tested if gender or social self-efficacy moderated the between- and within-person associations between loneliness and symptoms of anxiety and depression in the RI-CLPM. Preliminarily, we investigated RI-CLPMs of all groups without constraints and with time-invariant correlation and regression coefficients. If chi-square did not significantly deteriorate, we would keep the constraints in the moderation analyses. Lastly, we performed two multi-group analyses (one for gender and one for social self-efficacy) on the RI-CLPM with 1000 bootstrap replications and compared the parameters across groups using model constraints. We included gender as a time-invariant covariate in the social self-efficacy moderation model and social self-efficacy in the gender moderation model.

## Results

### Missing Data

When examining possible construct-level missingness in our data, we considered response rates across measurement occasions (Newman, 2014). See Table 1 for details. We also investigated the partial correlations of loneliness and missingness in loneliness at the following time point while controlling for the effect of symptoms of anxiety and depression (and vice versa) across measurement waves. There were no significant associations between a construct and following missingness in the same construct in either loneliness or anxiety and depression, indicating that the construct-level missingness in our data is approximate to or approaching missing at random (MAR) (Newman, 2014). We used full information maximum likelihood (FIML) estimation to handle potential construct-level missingness in our data.

**Table 1:** Response Rates

	T1	T2	T3	T4
Invited participants	1508	1508	1478	1478
Respondents	1151	1184	949	1016
Response rate	76.3%	78.5%	64.2%	68.7%
Full response rate	71.9%	74.7%	61.6%	66%
Partial response rate	4.4%	4.1%	2.6%	2.7%

*Full response rate* individuals who responded to both scales, *partial response rat*e individuals who responded to only one scale

### Descriptive Statistics and Correlations

The descriptive statistics of the study’s variables are presented in Table 2. The omega reliability results imply that symptoms of anxiety and depression and loneliness had satisfactory reliability across measurement occasions (*ω* > 0.80). The results imply that girls were considered a high-risk group concerning mental health at all time points (≥ 2.0), while the boys were not on any measurement occasion. Similarly, the group with low social self-efficacy reported values above the cut-off in symptoms of anxiety and depression throughout the study, while adolescents with high social self-efficacy did not. In general, boys and youths with high social self-efficacy experienced lower anxiety and depressive symptoms and loneliness compared to girls and youths with low social self-efficacy, respectively. According to Cohen (1988), the differences between genders and social self-efficacy groups were moderate (> 0.50) and small (> 0.20), respectively. Concerning loneliness, the differences between boys and girls were small, while the differences between the low and high social self-efficacy groups were large (> 0.80).

**Table 2:** Descriptive Statistics of the Study Variables

				Gender			Social self-efficacy		
				Boys	Girls		Low	High	
Variable	N	*ω*	Min – Max	*M (SD)*	*M (SD)*	*d*	*M (SD)*	*M (SD)*	*d*
AD symptoms T1	1114	0.90	1 – 4	1.55 (0.67)	2.00 (0.78)	–0.63	2.02 (0.82)	1.67 (0.69)	0.46
AD symptoms T2	1147	0.90	1 – 4	1.60 (0.63)	2.17 (0.82)	–0.76	2.15 (0.82)	1.78 (0.74)	0.47
AD symptoms T3	926	0.90	1 – 4	1.70 (0.71)	2.20 (0.80)	–0.65	2.19 (0.83)	1.84 (0.74)	0.46
AD symptoms T4	994	0.89	1 – 4	1.88 (0.28)	2.28 (0.81)	–0.51	2.35 (0.83)	1.94 (0.74)	0.53
Loneliness T1	1088	0.81	1 – 5	2.07 (0.72)	2.22 (0.78)	–0.20	2.52 (0.74)	1.88 (0.65)	0.94
Loneliness T2	1146	0.80	1 – 5	2.11 (0.70)	2.33 (0.78)	–0.31	2.62 (0.70)	1.94 (0.65)	1.03
Loneliness T3	915	0.81	1 – 5	2.20 (0.78)	2.38 (0.77)	–0.23	2.70 (0.71)	1.99 (0.68)	1.03
Loneliness T4	984	0.84	1 – 5	2.25 (0.84)	2.38 (0.80)	–0.17	2.71 (0.76)	2.00 (0.71)	0.96

*AD* anxiety and depressive, *d* cohen’s *d*

Tables 3 and 4 show the results of the correlations between the study’s variables across gender and social self-efficacy groups, respectively. The within-construct associations of loneliness and symptoms of anxiety and depression were positive and moderate (> 0.30) to large (> 0.50) (Cohen, 1988) across all time points in all subgroups. The between-construct correlations of loneliness and symptoms of anxiety and depression were positive and ranged from small (> 0.10) to large, wherein the effect sizes were smaller the greater the distances were in time. There were several correlations within and between loneliness and symptoms of anxiety and depression that were significantly larger for girls compared to boys (see Table 3 for details). Concerning social self-efficacy, only correlations within loneliness over time were significantly stronger within the low social self-efficacy group compared to the group with high social self-efficacy.

**Table 3:** Correlation Coefficients of the Study Variables Separated by Gender

	1.	2.	3.	4.	5.	6.	7.	8.
1. AD symptoms T1	–	0.64^a^	0.45	0.40	0.60	0.49^a^	0.41	0.27
2. AD symptoms T2	0.52	–	0.60	0.53	0.51^a^	0.62^a^	0.50	0.40
3. AD symptoms T3	0.49	0.57	–	0.66	0.28	0.45	0.61	0.43
4. AD symptoms T4	0.41	0.48	0.61	–	0.30	0.39	0.50	0.61
5. Loneliness T1	0.55	0.39	0.33	0.41	–	0.70^a^	0.52	0.44
6. Loneliness T2	0.33	0.52	0.41	0.42	0.59	–	0.68^a^	0.52
7. Loneliness T3	0.40	0.41	0.57	0.49	0.54	0.60	–	0.67
8. Loneliness T4	0.35	0.41	0.40	0.63	0.52	0.59	0.66	–

Boys are below the diagonal, and girls are above. All correlations are significant at the 0.001 level

*AD* anxiety and depressive

^a^significantly stronger correlation compared to the other subgroup

**Table 4:** Correlation Coefficients of the Study Variables Separated by Low and High Social Self-efficacy Groups

	1.	2.	3.	4.	5.	6.	7.	8.
1. AD symptoms T1	–	0.61	0.48	0.42	0.55	0.37	0.37	0.27
2. AD symptoms T2	0.63	–	0.61	0.53	0.46	0.54	0.42	0.35
3. AD symptoms T3	0.51	0.62	–	0.66	0.26	0.39	0.55	0.40
4. AD symptoms T4	0.43	0.52	0.64	–	0.31	0.37	0.42	0.58
5. Loneliness T1	0.56	0.42	0.29	0.27	–	0.54	0.38	0.41
6. Loneliness T2	0.45	0.59	0.41	0.35	0.64^a^	–	0.52	0.48
7. Loneliness T3	0.36	0.44	0.58	0.49	0.49^a^	0.62^a^	–	0.59
8. Loneliness T4	0.23	0.30	0.37	0.32	0.32	0.42	0.60	–

The low social self-efficacy group is below the diagonal, and the high social self-efficacy group is above. All correlations are significant at the 0.001 level

*AD* anxiety and depressive

^a^significantly stronger correlation compared to the other subgroup

### The Longitudinal Association Between Loneliness and Symptoms of Anxiety and Depression

**Fig. 2:**
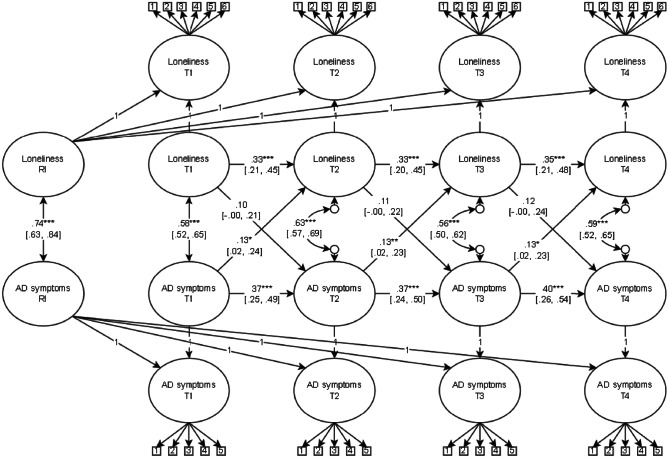
Random Intercept Cross-lagged Panel Model of Loneliness and Symptoms of Anxiety and Depression. *Note*. RI = random intercept, AD = anxiety and depressive. Standardised estimates presented with 95% confidence interval in brackets. *** *p* < 0.001, **p < 0.01, *p < 0.05

The RI-CLPM of loneliness and symptoms of anxiety and depression with time-invariant covariates and metric longitudinal invariance constraints achieved acceptable model fit (CFI > 0.90, RMSEA < 0.08, SRMR < 0.08). The model fit did not significantly deteriorate when we applied time-invariant constraints on the correlation and regression coefficients (Δχ^2^ = 8.28, Δ*df* = 11, *p* = 0.688): χ^2^ = 2677.38 *df* = 1101, *p* < 0.001, RMSEA = 0.04, 95% CI [0.03, 0.04], CFI = 0.94, SRMR = 0.05. Therefore, the time-invariance constraints were considered tenable. The standardised model results are presented in Fig. 2, and more details are provided in Appendix C.

At the between-person level, the random intercepts of loneliness and symptoms of anxiety and depression were positively and strongly related. This indicates that individuals who, in general, experienced high levels of loneliness during mid-late adolescence also reported high levels of anxiety and depression during the same time. At the within-person level, there were positive and strong associations between loneliness and symptoms of anxiety and depression at each time point. This implies that positive or negative fluctuations at one time point in loneliness are related to similar fluctuations in symptoms of anxiety and depression simultaneously. We found positive carry-over stability effects in both loneliness and symptoms of anxiety and depression over time. This implies that adolescents who experienced higher or lower levels than expected of either loneliness or symptoms of anxiety and depression on one occasion had an increased likelihood of experiencing the same deviation in the corresponding construct at the next time point. Lastly, there were positive and significant cross-lagged effects from symptoms of anxiety and depression to later loneliness, but not the other way around. This indicates that adolescents who experienced a deviation in anxiety and depressive symptoms at one time point likely experienced the same deviation in loneliness at the next time point but not vice versa.

### Moderation of the Association Between Loneliness and Symptoms of Anxiety and Depression

#### Gender

**Fig. 3:**
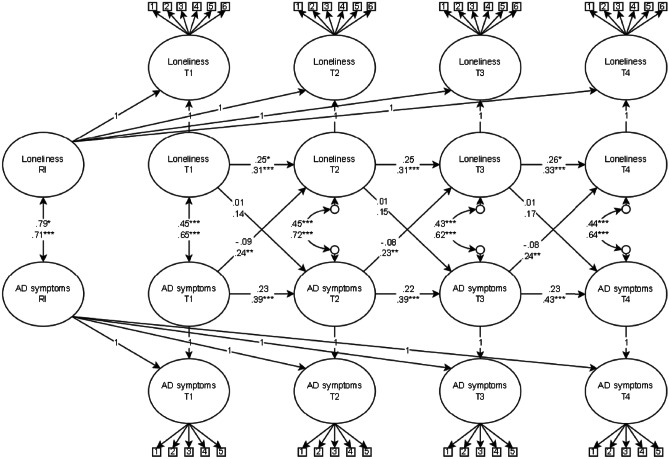
Random Intercept Cross-lagged Panel Model of Loneliness and Symptoms of Anxiety and Depression Moderated by Gender, *Note*. RI = random intercept, AD = anxiety and depressive. Boys on upper line and girls on lower line. Standardised estimates presented in figure. *** *p* < 0.001, **p < 0.01, *p < 0.05

When comparing unconstrained to constrained models within each gender, the model fit did not significantly deteriorate for either girls or boys (*p* > 0.05). Thus, we tested the difference between six parameters between boys and girls in the RI-CLPM of loneliness and symptoms of anxiety and depression: the time-invariant correlations, autoregressive and cross-lagged regression coefficients at the within-person level and the correlation between the random intercepts. The gender multi-group RI-CLPM of loneliness and symptoms of anxiety and depression achieved satisfactory fit: χ^2^ = 4065.90, *df* = 2163, *p* < 0.001, RMSEA = 0.04, 95% CI [0.04, 0.04], CFI = 0.92, SRMR = 0.06. The model results are presented in Fig. 3, and further details are provided in Appendix D. We found that gender differed on two parameters. One, the concurrent state-like associations between loneliness and symptoms of anxiety and depression were significantly stronger for girls across all measurement waves compared to boys (*r*_diff_ = 0.07, *p* < 0.05). Two, the within-person effects from symptoms of anxiety and depression to later loneliness throughout the study were significantly higher for girls compared to boys (B_diff_ = 0.30, *p* < 0.001).

#### Social Self-efficacy

**Fig. 4:**
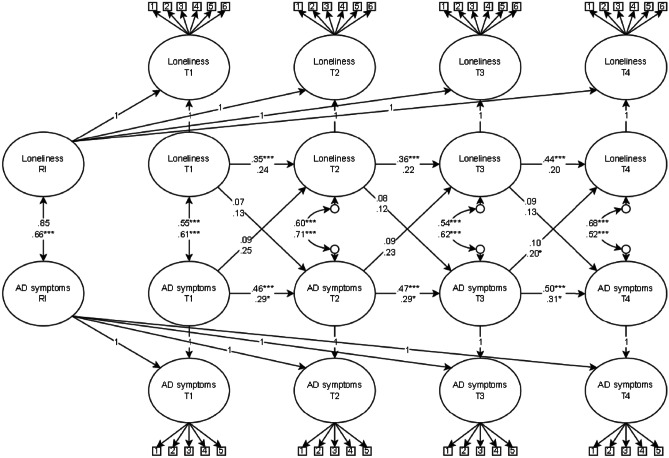
Random Intercept Cross-lagged Panel Model of Loneliness and Symptoms of Anxiety and Depression Moderated by Social Self-efficacy. *Note*. RI = random intercept, AD = anxiety and depressive. High social self-efficacy on upper line and low social self-efficacy on lower line. Standardised estimates presented in figure. *** *p* < 0.001, *p < 0.05

The model fit did not significantly change when comparing nested, fully time-invariant models to completely unconstrained models within the low and high social self-efficacy groups (*p* > 0.05). As such, we tested the difference between the same six parameters as in the gender moderation model: the time-invariant correlations, autoregressive and cross-lagged regression coefficients at the within-person level and the correlation between the random intercepts. The results from the social self-efficacy moderation analysis on the loneliness and symptoms of anxiety and depression RI-CLPM produced acceptable model fit: χ^2^ = 4002.76, *df* = 2163, *p* < 0.001, RMSEA = 0.04, 95% CI [0.04, 0.04], CFI = 0.91, SRMR = 0.07. The standardised results from the model are presented in Fig. 4, and unstandardised and standardised estimates, standard errors, and bootstrapped 95% confidence intervals are presented in Appendix E. Even though the low and high social self-efficacy groups have somewhat different coefficients, these differences were not significant (*p* > 0.05). This indicates that high social self-efficacy does not act as a protective factor in the within- and between-person association between loneliness and symptoms of anxiety and depression.

## Discussion

The present study had two main goals. First, we wanted to investigate how loneliness and symptoms of anxiety and depression were associated at the within- and between-person level during mid-late adolescence. Second, gender and social self-efficacy were tested as possible moderators in this relationship. We found that symptoms of anxiety and depression had an effect on later loneliness but not vice versa. Next, girls seemed to be more sensitive and at risk regarding the state-like associations between loneliness and symptoms of anxiety and depression compared to boys. Lastly, social self-efficacy might not be considered an important protective factor in the relationship between loneliness and symptoms of anxiety and depression.

### Loneliness and Symptoms of Anxiety and Depression in a Developmental Perspective

#### The Trait-like Association

In support of hypothesis 1, our results indicate that adolescents who, in general, experience symptoms of anxiety and depression are highly likely to also feel lonely during three years in the mid-late adolescent period. In other words, the trait-like components of anxiety and depression symptoms and loneliness were strongly related. This is largely in line with previous research, which has found positive associations between loneliness, anxiety, and depression (Beutel et al., 2017; Erzen & Çikrikci, 2018; Park et al., 2020). The trait-like association between loneliness and symptoms of anxiety and depression might, in part, be related to the conceptualisation of the constructs. For example, Peplau and Perlman (1982) illustrate that loneliness taps into feelings that are central to anxiety and depression, such as distress, sadness, lacking care, psychological discomfort, and boredom. Individuals who feel lonely on a trait level are more likely to be less socially competent, believe their loneliness is due to ever-lasting personal qualities, and struggle to overcome social shortages (Perlman & Peplau, 1998). Adolescents with these perceptions and beliefs are arguably expected also to experience feelings of sadness, apprehension, worry, nervousness, and hopelessness simultaneously. This is further supported by the strong state-like associations between loneliness and symptoms of anxiety and depression across time. Consistently in mid-late adolescence, individuals that were lonelier than usual on one occasion, were increasingly likely to experience higher than average symptoms of anxiety and depression at the same time point.

#### Carry-over Stability Effects

The positive carry-over stability effects in loneliness might be related to the major social transitions that take place during mid-late adolescence. First, during adolescence, peer relationships become increasingly important, while parental influence diminishes (Larson & Richards, 1991; Prinstein & Dodge, 2008), causing several social expectancies and alterations that can be challenging (or exceedingly easy) for some individuals. Second, most people begin an upper secondary school education at age 15–16, which is a major shift in adolescents’ social lives (Eccles & Roeser, 2009, 2011; Wigfield et al., 1991). Third, during adolescence, individuals experience sexual maturation and an increased interest in pursuing romantic relationships with others (Collins et al., 2009), which could both alter individuals’ perception of what being alone means and bring about several time periods of unusual relationship satisfaction or dissatisfaction.

We found positive carry-over stability effects within symptoms of anxiety and depression, implying that adolescents who experienced an unusual level of anxiety and depression at one time point likely experienced the same unusual level one year later. This finding has been discussed in light of helplessness-hopelessness theory (Kristensen et al., 2021), which states that periods of increased symptoms of anxiety and depression are likely to be followed by the same deviations of anxiety and depressive symptoms due to a vicious cycle of exacerbating symptoms over time (Alloy et al., 1990).

#### The Temporal Relationship between Loneliness and Symptoms of Anxiety and Depression

In contradiction to hypothesis 2 and previous research (see Cacioppo & Cacioppo, 2018 for an overview; Lim et al., 2016; Wei et al., 2005), our results indicate that feelings of unusually high or low loneliness did not predict unexpectedly high or low symptoms of later anxiety and depression. Because previous research has largely failed to separate the within-person effects from between-person effects, the assumption that loneliness increases subsequent mental health issues might be overestimated (Hamaker et al., 2015). Thus, although reducing feelings of being socially isolated is an important goal in itself, our findings question whether modifying unusual perceptions of social isolation would improve unexpected levels of adolescent anxiety and depressive symptoms. Interventions aimed at reducing loneliness have not separated implementation strategies between persistent (trait) and transient (state) loneliness (Eccles & Qualter, 2021), which could be important in future endeavours aimed at decreasing unusual loneliness.

We found that higher-than-normal symptoms of anxiety and depression were predictive of unusually high feelings of subsequent loneliness throughout the study. This result could imply that initiatives aimed at reducing anxiety and depressive symptoms in adolescence (see e.g., Das et al., 2016 for an overview) may decrease loneliness as a result. However, it is also important to identify adolescents who experience symptoms of anxiety and depression that are out of the ordinary (i.e., deviating from their personal norm). Significant adults that are close to adolescents, such as parents (Logan & King, 2001), teachers (Rothì et al., 2008), school healthcare professionals (Levinson et al., 2019), and guidance counsellors (Collins, 2014) or school administrators (Green et al., 2013) need resources and competence to identify and deal with negative fluctuations in young people’s mental health to avoid unusual escalations of loneliness.

### Is the Association between Loneliness and Symptoms of Anxiety and Depression More Salient for Some?

In support of hypothesis 4, we found that 1) the state-like association between loneliness and symptoms of anxiety and depression and 2) the within-person effect of anxiety and depressive symptoms on later loneliness were more salient for girls than boys. Girls rely on social relationships and the support these bring about to a greater extent (Derdikman-Eiron et al., 2012). Because unexpected loneliness and symptoms of anxiety and depression arise simultaneously for girls, they could have increased difficulties in seeking help due to a perception of not having caring and supportive relationships (Gadalla, 2008; Gagné et al., 2014) or an unease about opening up regarding their loneliness (Verity et al., 2022). Girls’ mental health benefits more from social bonds and support compared to boys (Rose & Rudolph, 2006). Thus, if lonely girls believe they do not have a socially supportive environment, they could have more trouble seeking help for their symptoms of anxiety and depression, resulting in a reinforcing effect of anxiety and depressive symptoms on later loneliness experiences.

In contradiction to hypothesis 5, we found that social self-efficacy did not moderate any effects in the association between loneliness and symptoms of anxiety and depression. The lack of moderation is somewhat surprising, considering theories and models within a vulnerability framework (e.g., Hankin & Abela, 2005) argue that low social self-efficacy functions as a vulnerability factor in the development of anxiety and depressive symptoms (Bandura, 1997). The results indicate that adolescents likely experience loneliness following an unexpected rise in anxiety and depression symptoms, despite how socially capable they perceive themselves to be. As such, even though people who are socially efficacious behave in ways that are socially desirable, they could still lack fundamental care and understanding from the people around them. It is possible that a central element to this (non)effect is that the experience of loneliness is a qualitative issue, not quantitative – we need connections that satisfy our need to belong (e.g., Baumeister & Leary, 1995; Bowlby, 1979; Cacioppo & Patrick, 2008; Ryan & Deci, 2017), not necessarily several connections that do not (Qualter et al., 2015).

### Limitations

One limitation of the current study is the dichotomisation of the social self-efficacy variable. This could lead to several problems regarding power and inferences. First, by creating a dummy variable, when said variable is continuous in nature, we assume that the logical cut-off point is the median level without really knowing this to be true. Second, we lose statistical power by the dichotomisation (Cohen, 1983; MacCallum et al., 2002). Third, there is a chance that we miss non-linearity in the relationship between the dichotomised variable and other factors. However, despite the drawbacks of creating a dummy variable of social self-efficacy, the statistical benefits of using the factor as a multi-group moderator variable in the within-person association between loneliness and symptoms of anxiety and depression were deemed to outweigh the downsides.

## Supplementary Information

Below is the link to the electronic supplementary material.

Supplementary file1 (DOCX 239 KB)

Supplementary file2 (DOCX 18 KB)

Supplementary file3 (DOCX 18 KB)

Supplementary file4 (DOCX 22 KB)

Supplementary file5 (DOCX 22 KB)
