# Supplementary material for: Hello Darkness, My Old Friend: Moderating a Random Intercept Cross-lagged Panel Model of Loneliness and Symptoms of Anxiety and Depression
Source: Res Child Adolesc Psychopathol. 2022 Nov 23;51(3):383–97. doi: 10.1007/s10802-022-00995-1 (PMC9908696; doi:10.1007/s10802-022-00995-1)
Supplement: Supplementary file 1 — Supplementary file1 (DOCX 239 KB) [file 10802_2022_995_MOESM1_ESM.docx]

**Appendix A**

**Developmentally Based Interpersonal Model of Youth Depression**


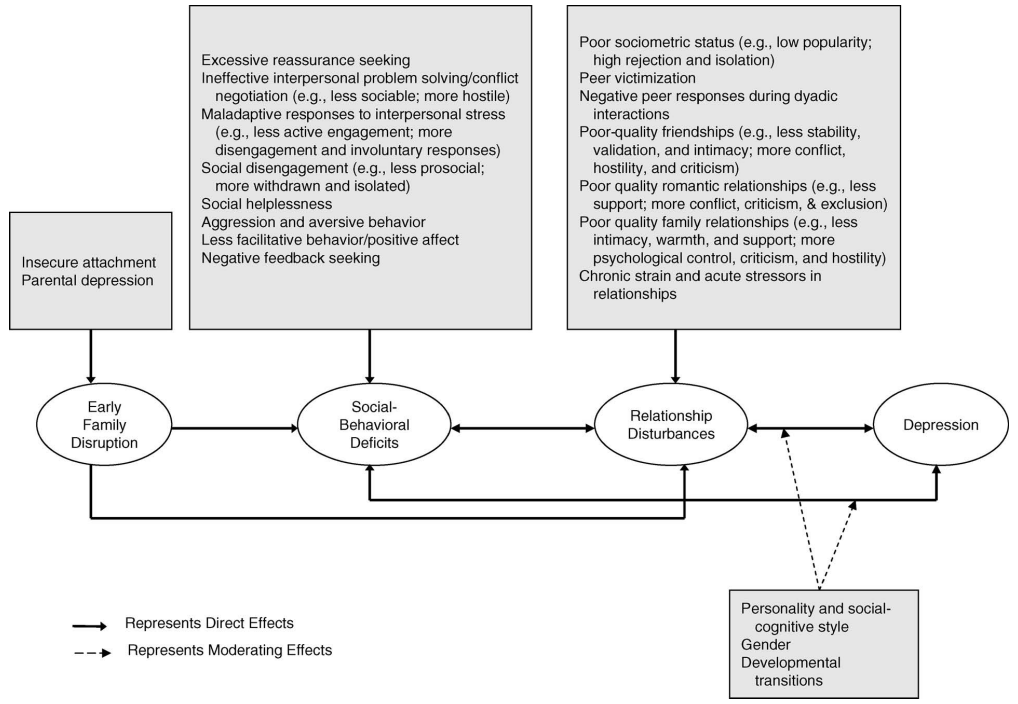


*Note*. Model from Rudolph et al. (2008, p. 80).
