# Supplementary material for: Hello Darkness, My Old Friend: Moderating a Random Intercept Cross-lagged Panel Model of Loneliness and Symptoms of Anxiety and Depression
Source: Res Child Adolesc Psychopathol. 2022 Nov 23;51(3):383–97. doi: 10.1007/s10802-022-00995-1 (PMC9908696; doi:10.1007/s10802-022-00995-1)
Supplement: Supplementary file 2 — Supplementary file2 (DOCX 18 KB) [file 10802_2022_995_MOESM2_ESM.docx]

**Appendix B**

**Measurement Invariance**

|  | *χ*^2^ | *df* | RMSEA [90% CI] | CFI | SRMR | ΔRMSEA | ΔCFI | ΔSRMR | |
| --- | --- | --- | --- | --- | --- | --- | --- | --- | --- |
| **Across time** | | | | | | | | | |
| *Loneliness* | | | | | | | | |  |
| Configural | 779.386 | 210 | .043 [.039, .046] | .952 | .052 |  |  |  | |
| Metric | 841.137 | 228 | .042 [.039, .046] | .949 | .058 | .001 | .003 | .006 | |
| *Symptoms of anxiety and depression* | | | | | | | | |  |
| Configural | 428.732 | 134 | .038 [.034, .042] | .978 | .027 |  |  |  | |
| Metric | 466.374 | 149 | .038 [.034, .042] | .976 | .034 | .000 | .002 | .007 | |
| **Across Time and Social Self-efficacy Groups** | | | | | | | | | |
| *Loneliness* | | | | | | | | |  |
| Configural | 977.165 | 420 | .042 [.039, .046] | .943 | .055 |  |  |  | |
| Metric | 1025.850 | 444 | .042 [.039, .045] | .941 | .061 | .000 | .002 | .006 | |
| *Symptoms of anxiety and depression* | | | | | | | | |  |
| Configural | 591.293 | 268 | .040 [.036, .045] | .974 | .033 |  |  |  | |
| Metric^a^ | 635.293 | 287 | .040 [.036, .045] | .972 | .061 | .000 | .002 | .028 | |
| **Across Time and Gender** | | | | | | | | | |
| *Loneliness* | | | | | | | | |  |
| Configural | 1025.549 | 420 | .044 [.041, .047] | .949 | .059 |  |  |  | |
| Metric | 1082.530 | 444 | .044 [.041, .047] | .946 | .063 | .000 | .003 | .004 | |
| *Symptoms of anxiety and depression* | | | | | | | | |  |
| Configural | 615.975 | 268 | .042 [.037, .046] | .971 | .039 |  |  |  | |
| Metric^a^ | 663.697 | 287 | .042 [.038, .046] | .969 | .066 | .000 | .002 | .027 | |

*Note*. ^a^ = one indicator factor loading constraint removed for model fit.
