# Supplementary material for: Hello Darkness, My Old Friend: Moderating a Random Intercept Cross-lagged Panel Model of Loneliness and Symptoms of Anxiety and Depression
Source: Res Child Adolesc Psychopathol. 2022 Nov 23;51(3):383–97. doi: 10.1007/s10802-022-00995-1 (PMC9908696; doi:10.1007/s10802-022-00995-1)
Supplement: Supplementary file 3 — Supplementary file3 (DOCX 18 KB) [file 10802_2022_995_MOESM3_ESM.docx]

**Appendix C**

**Standardised and Unstandardised Estimates, Standard Error, and 95% Confidence Intervals from the RI-CLPM of Loneliness and Symptoms of Anxiety and Depression**

|  |  |  | Unstandardised | | | Standardised | | |
| --- | --- | --- | --- | --- | --- | --- | --- | --- |
|  |  |  | Est. | SE | 95% CI | Est. | SE | 95% CI |
| *Carry-over stability effects* | | | | | | | | |
| T1 AD symptoms | → | T2 AD symptoms | .385 | .067 | .255, .516 | .370 | .061 | .251, .490 |
| T2 AD symptoms | → | T3 AD symptoms | .385 | .067 | .255, .516 | .371 | .066 | .242, .500 |
| T3 AD symptoms | → | T4 AD symptoms | .385 | .067 | .255, .516 | .398 | .073 | .255, .541 |
| T1 Loneliness | → | T2 Loneliness | .355 | .068 | .222, .487 | .326 | .061 | .207, .445 |
| T2 Loneliness | → | T3 Loneliness | .355 | .068 | .222, .487 | .326 | .064 | .200, .452 |
| T3 Loneliness | → | T4 Loneliness | .355 | .068 | .222, .487 | .347 | .069 | .213, .482 |
| *Cross-lagged effects* | | | | | | | | |
| T1 AD symptoms | → | T2 Loneliness | .121 | .050 | .024, .218 | .132 | .055 | .024, .241 |
| T2 AD symptoms | → | T3 Loneliness | .121 | .050 | .024, .218 | .126 | .053 | .022, .231 |
| T3 AD symptoms | → | T4 Loneliness | .121 | .050 | .024, .218 | .128 | .054 | .023, .234 |
| T1 Loneliness | → | T2 AD symptoms | .127 | .064 | .002, .252 | .103 | .054 | –.002, .208 |
| T2 Loneliness | → | T3 AD symptoms | .127 | .064 | .002, .252 | .108 | .056 | –.002, .218 |
| T3 Loneliness | → | T4 AD symptoms | .127 | .064 | .002, .252 | .121 | .062 | –.001, .244 |
| *Correlations* | | | | | | | | |
| T1 Loneliness | ↔ | T1 AD symptoms | .137 | .008 | .121, .153 | .582 | .032 | .519, .645 |
| T2 Loneliness | ↔ | T2 AD symptoms | .137 | .008 | .121, .153 | .630 | .032 | .567, .692 |
| T3 Loneliness | ↔ | T3 AD symptoms | .137 | .008 | .121, .153 | .563 | .031 | .503, .623 |
| T4 Loneliness | ↔ | T4 AD symptoms | .137 | .008 | .121, .153 | .587 | .032 | .523, .650 |
| RI Loneliness | ↔ | RI AD symptoms | .122 | .017 | .089, .154 | .737 | .054 | .632, .842 |

*Note*. AD = anxiety and depressive, RI = random intercept, Est. = estimate, SE = standard error, CI = confidence interval.
