# Supplementary material for: Hello Darkness, My Old Friend: Moderating a Random Intercept Cross-lagged Panel Model of Loneliness and Symptoms of Anxiety and Depression
Source: Res Child Adolesc Psychopathol. 2022 Nov 23;51(3):383–97. doi: 10.1007/s10802-022-00995-1 (PMC9908696; doi:10.1007/s10802-022-00995-1)
Supplement: Supplementary file 4 — Supplementary file4 (DOCX 22 KB) [file 10802_2022_995_MOESM4_ESM.docx]

**Appendix D**

**Standardised and Unstandardised Estimates, Standard Error, and 95% Confidence Intervals from the RI-CLPM of Loneliness and Symptoms of Anxiety and Depression Moderated by Gender**

|  |  |  | Unstandardised | | | Standardised | | |
| --- | --- | --- | --- | --- | --- | --- | --- | --- |
|  |  |  | Est. | SE | 95% CI | Est. | SE | 95% CI |
| **Boys** | | | | | | | | |
| *Carry over-stability effects* | | | | | | | | |
| T1 AD symptoms | → | T2 AD symptoms | .219 | .162 | –.104, .520 | .226 | .163 | –.114, .511 |
| T2 AD symptoms | → | T3 AD symptoms | .219 | .162 | –.104, .520 | .215 | .159 | –.100, .528 |
| T3 AD symptoms | → | T4 AD symptoms | .219 | .162 | –.104, .520 | .227 | .173 | –.095, .569 |
| T1 Loneliness | → | T2 Loneliness | .264 | .128 | .028, .537 | .245 | .118 | .026, .513 |
| T2 Loneliness | → | T3 Loneliness | .264 | .128 | .028, .537 | .252 | .133 | .031, .570 |
| T3 Loneliness | → | T4 Loneliness | .264 | .128 | .028, .537 | .263 | .130 | .023, .546 |
| *Cross-lagged effects* | | | | | | | | |
| T1 AD symptoms | → | T2 Loneliness | –.083 | .096 | –.273, .110 | –.085 | .096 | –.266, .112 |
| T2 AD symptoms | → | T3 Loneliness | –.083 | .096 | –.273, .110 | –.079 | .089 | –.236, .109 |
| T3 AD symptoms | → | T4 Loneliness | –.083 | .096 | –.273, .110 | –.080 | .092 | –.246, .104 |
| T1 Loneliness | → | T2 AD symptoms | .011 | .114 | –.224, .236 | .010 | .107 | –.184, .231 |
| T2 Loneliness | → | T3 AD symptoms | .011 | .114 | –.224, .236 | .011 | .112 | –.185, .248 |
| T3 Loneliness | → | T4 AD symptoms | .011 | .114 | –.224, .236 | .011 | .119 | –.203, .275 |
| *Correlations* | | | | | | | | |
| T1 Loneliness | ↔ | T1 AD symptoms | .088 | .015 | .061, .116 | .447 | .084 | .295, .613 |
| T2 Loneliness | ↔ | T2 AD symptoms | .088 | .015 | .061, .116 | .452 | .069 | .326, .596 |
| T3 Loneliness | ↔ | T3 AD symptoms | .088 | .015 | .061, .116 | .425 | .081 | .287, .603 |
| T4 Loneliness | ↔ | T4 AD symptoms | .088 | .015 | .061, .116 | .440 | .082 | .294, .621 |
| RI Loneliness | ↔ | RI AD symptoms | .130 | .023 | .080, .170 | .794 | .349 | .607, 1.212 |
| **Girls** | | | | | | | | |
| *Carry-over stability effects* | | | | | | | | |
| T1 AD symptoms | → | T2 AD symptoms | .417 | .096 | .219, .594 | .389 | .087 | .203, .550 |
| T2 AD symptoms | → | T3 AD symptoms | .417 | .096 | .219, .594 | .389 | .093 | .200, .567 |
| T3 AD symptoms | → | T4 AD symptoms | .417 | .096 | .219, .594 | .425 | .102 | .212, .610 |
| T1 Loneliness | → | T2 Loneliness | .342 | .088 | .169, .509 | .309 | .078 | .162, .455 |
| T2 Loneliness | → | T3 Loneliness | .342 | .088 | .169, .509 | .310 | .083 | .153, .477 |
| T3 Loneliness | → | T4 Loneliness | .342 | .088 | .169, .509 | .331 | .086 | .168, .496 |
| *Cross-lagged effects* | | | | | | | | |
| T1 AD symptoms | → | T2 Loneliness | .215 | .073 | .066, .352 | .235 | .081 | .074, .387 |
| T2 AD symptoms | → | T3 Loneliness | .215 | .073 | .066, .352 | .228 | .079 | .072, .383 |
| T3 AD symptoms | → | T4 Loneliness | .215 | .073 | .066, .352 | .237 | .082 | .074, .400 |
| T1 Loneliness | → | T2 AD symptoms | .186 | .102 | –.018, .374 | .143 | .082 | –.015, .307 |
| T2 Loneliness | → | T3 AD symptoms | .186 | .102 | –.018, .374 | .148 | .083 | –.015, .307 |
| T3 Loneliness | → | T4 AD symptoms | .186 | .102 | –.018, .374 | .166 | .091 | –.017, .334 |
| *Correlations* | | | | | | | | |
| T1 Loneliness | ↔ | T1 AD symptoms | .159 | .011 | .136, .181 | .646 | .042 | .558, .720 |
| T2 Loneliness | ↔ | T2 AD symptoms | .159 | .011 | .136, .181 | .720 | .045 | .635, .816 |
| T3 Loneliness | ↔ | T3 AD symptoms | .159 | .011 | .136, .181 | .621 | .049 | .536, .720 |
| T4 Loneliness | ↔ | T4 AD symptoms | .159 | .011 | .136, .181 | .642 | .042 | .563, .726 |
| RI Loneliness | ↔ | RI AD symptoms | .121 | .023 | .077, .170 | .705 | .086 | .548, .878 |

*Note*. AD = anxiety and depressive, RI = random intercept, Est. = estimate, SE = standard error, CI = confidence interval.
