# Supplementary material for: Hello Darkness, My Old Friend: Moderating a Random Intercept Cross-lagged Panel Model of Loneliness and Symptoms of Anxiety and Depression
Source: Res Child Adolesc Psychopathol. 2022 Nov 23;51(3):383–97. doi: 10.1007/s10802-022-00995-1 (PMC9908696; doi:10.1007/s10802-022-00995-1)
Supplement: Supplementary file 5 — Supplementary file5 (DOCX 22 KB) [file 10802_2022_995_MOESM5_ESM.docx]

**Appendix E**

**Standardised and Unstandardised Estimates, Standard Error, and 95% Confidence Intervals from the RI-CLPM of Loneliness and Symptoms of Anxiety and Depression Moderated by Social Self-efficacy**

|  |  |  | Unstandardised | | | Standardised | | |
| --- | --- | --- | --- | --- | --- | --- | --- | --- |
|  |  |  | Est. | SE | 95% CI | Est. | SE | 95% CI |
| **Low social self-efficacy** | | | | | | | | |
| *Carry-over stability effects* | | | | | | | | |
| T1 AD symptoms | → | T2 AD symptoms | .300 | .131 | .051, .554 | .292 | .124 | .049, .524 |
| T2 AD symptoms | → | T3 AD symptoms | .300 | .131 | .051, .554 | .286 | .125 | .049, .529 |
| T3 AD symptoms | → | T4 AD symptoms | .300 | .131 | .051, .554 | .308 | .138 | .051, .585 |
| T1 Loneliness | → | T2 Loneliness | .244 | .145 | –.039, .533 | .243 | .136 | –.047, .495 |
| T2 Loneliness | → | T3 Loneliness | .244 | .145 | –.039, .533 | .224 | .138 | –.033, .499 |
| T3 Loneliness | → | T4 Loneliness | .244 | .145 | –.039, .533 | .204 | .126 | –.031, .472 |
| *Cross-lagged effects* | | | | | | | | |
| T1 AD symptoms | → | T2 Loneliness | .201 | .101 | –.014, .398 | .246 | .132 | –.016, .507 |
| T2 AD symptoms | → | T3 Loneliness | .201 | .101 | –.014, .398 | .231 | .122 | –.014, .478 |
| T3 AD symptoms | → | T4 Loneliness | .201 | .101 | –.014, .398 | .202 | .102 | –.014, .393 |
| T1 Loneliness | → | T2 AD symptoms | .158 | .140 | –.152, .413 | .125 | .112 | –.116, .333 |
| T2 Loneliness | → | T3 AD symptoms | .158 | .140 | –.152, .413 | .120 | .111 | –.114, .331 |
| T3 Loneliness | → | T4 AD symptoms | .158 | .140 | –.152, .413 | .134 | .121 | –.122, .360 |
| *Correlations* | | | | | | | | |
| T1 Loneliness | ↔ | T1 AD symptoms | .143 | .014 | .115, .170 | .610 | .055 | .504, .700 |
| T2 Loneliness | ↔ | T2 AD symptoms | .143 | .014 | .115, .170 | .710 | .067 | .591, .859 |
| T3 Loneliness | ↔ | T3 AD symptoms | .143 | .014 | .115, .170 | .618 | .065 | .503, .755 |
| T4 Loneliness | ↔ | T4 AD symptoms | .143 | .014 | .115, .170 | .522 | .057 | .408, .641 |
| RI Loneliness | ↔ | RI AD symptoms | .140 | .028 | .086, .199 | .658 | .110 | .512, .855 |
| **High social self-efficacy** | | | | | | | | |
| *Carry-over stability effects* | | | | | | | | |
| T1 AD symptoms | → | T2 AD symptoms | .480 | .120 | .235, .690 | .461 | .113 | .232, .654 |
| T2 AD symptoms | → | T3 AD symptoms | .480 | .120 | .235, .690 | .465 | .123 | .220, .688 |
| T3 AD symptoms | → | T4 AD symptoms | .480 | .120 | .235, .690 | .500 | .128 | .239, .722 |
| T1 Loneliness | → | T2 Loneliness | .395 | .090 | .213, .579 | .352 | .086 | .181, .532 |
| T2 Loneliness | → | T3 Loneliness | .395 | .090 | .213, .579 | .362 | .088 | .193, .535 |
| T3 Loneliness | → | T4 Loneliness | .395 | .090 | .213, .579 | .437 | .094 | .241, .614 |
| *Cross-lagged effects* | | | | | | | | |
| T1 AD symptoms | → | T2 Loneliness | .090 | .075 | –.064, .236 | .094 | .077 | –.066, .241 |
| T2 AD symptoms | → | T3 Loneliness | .090 | .075 | –.064, .236 | .089 | .076 | –.062, .241 |
| T3 AD symptoms | → | T4 Loneliness | .090 | .075 | –.064, .236 | .102 | .087 | –.066, .282 |
| T1 Loneliness | → | T2 AD symptoms | .089 | .094 | –.090, .271 | .073 | .082 | –.068, .249 |
| T2 Loneliness | → | T3 AD symptoms | .089 | .094 | –.090, .271 | .080 | .087 | –.077, .252 |
| T3 Loneliness | → | T4 AD symptoms | .089 | .094 | –.090, .271 | .091 | .096 | –.088, .277 |
| *Correlations* | | | | | | | | |
| T1 Loneliness | ↔ | T1 AD symptoms | .134 | .013 | .105, .158 | .552 | .061 | .431, .672 |
| T2 Loneliness | ↔ | T2 AD symptoms | .134 | .013 | .105, .158 | .600 | .053 | .498, .702 |
| T3 Loneliness | ↔ | T3 AD symptoms | .134 | .013 | .105, .158 | .543 | .060 | .437, .663 |
| T4 Loneliness | ↔ | T4 AD symptoms | .134 | .013 | .105, .158 | .675 | .053 | .577, .786 |
| RI Loneliness | ↔ | RI AD symptoms | .100 | .026 | .048, .149 | .852 | .471 | .627, 1.909 |

*Note*. AD = anxiety and depressive, RI = random intercept, Est. = estimate, SE = standard error, CI = confidence interval.
